# The complete mitochondrial genome of *Bombus filchnerae* (Hymenoptera: Apidae) and phylogenetic analysis

**DOI:** 10.1080/23802359.2021.1966329

**Published:** 2021-08-24

**Authors:** Feng Zhou, Guoyan Zhang, Liyuan Yao, Peng Yu, Lingyun Chen, Yingzhi Ning

**Affiliations:** College of Life Science, Northwest Normal University, Lanzhou, China

**Keywords:** Bumblebee, mitochondrial genome, phylogenetic relationship

## Abstract

Here, we report the complete mitochondrial genome (mitogenome) of *Bombus filchnerae* (Hymenoptera: Apidae). The genome size of *B. filchnerae* was 18,553 bp with 88.7% A + T content, containing 13 protein-coding genes (PCGs), 22 tRNA genes, two rRNA genes, and an AT-rich control region (D-loop). tRNA rearrangement was observed in this mitochondrial genome when compared to those of other bumblebee species (e.g. *Bombus breviceps* and *Bombus asiaticus*). All the 13 PCGs initiated with typical ATN codons. Among them, 11 PCGs terminated with TAA, only *nad4* and *nad5* with incomplete stop codon TA and T, respectively. All the 22 tRNAs can be folded into typical cloverleaf structure, except for *trnS1*, whose dihydrouridine (DHU) arm forms a simple loop. The phylogenetic analysis based on the concatenated nucleotide sequences of all 13 PCGs indicated that *B. filchnerae* showed the closest relationship with *Bombus pascuorum*, forming a mono clade of the subgenus *Thoracobombus*, with well-resolved relationships among nine *Bombus* subgenera.

Bumblebees (Hymenoptera: Apidae) are important pollinators for wild plants and greenhouses crops (Velthuis and van Doorn 2006). The bumblebee group consists of about 250 known species subdivided into 15 subgenera (Williams et al. 2008). *Bombus filchnerae* was the species belonging to the subgenus *Thoracobombus*, and widely distributed in North China with elevations of 602–2659 m (An et al. 2014). Until now, the complete mitochondrial genome of *B. filchnerae* has not been reported. Here, we first sequenced and characterized the complete mitochondrial genome of *B. filchnerae* and further tested the phylogenetic relationships combining with other available bumblebee mitogenomes retrieved from the GenBank database.

Female bumblebees of *B. filchnerae* were collected from Qilianshan nature reserve in Qinghai province, China (38°5′19″N, 100°21′8″E). Specimens were stored in the Institute of Zoology and Ecology, College of Life Science, Northwest Normal University, Lanzhou, China (accession number: QL2020006). The genomic DNA was extracted from thoracic muscle of a single specimen, which was sequenced by Illumina NovaSeq 6000 platform with both directions of 150 bp reads. The MITObim v1.9.1 (Hahn et al. 2013) was used to assemble the mitogenome based on 6 Gb clean data. The assembled mitogenome was annotated using the MITOS web server (Bernt et al. 2013) under the invertebrate mitochondrial code. The tRNA genes were confirmed by ARWEN online application (Laslett and Canback 2008). The phylogenetic analysis was performed by the IQ-TREE 2 software (Minh et al. 2020). The newly determined genome from the present study was deposited in the GenBank database (accession number: MW741886.2).

The complete mitogenome of *B. filchnerae* was 18,553 bp in length. The A + T content of the whole sequenced genome was 88.7% (44.0% A, 44.7% T, 6.3% C, and 5.0% G), indicating significant A + T bias. This mitogenome contained 13 protein-coding genes (PCGs), 22 tRNA genes, two rRNA genes, and an AT-rich control region (D-loop). Most genes showed an identical order with other bumblebee species (e.g. *Bombus breviceps* and *Bombus asiaticus*) (Zhao et al. 2017; Zhao et al. 2019), except for reversed direction of tRNA genes: *trnA* to *trnM*, *trnN* to *trnR*, and *trnT* to *trnP* (from 5′ to 3′), which was similar to those of *Bombus pascuorum* (accession number: KT164630.1). Like other bumblebees (Du et al. 2016), all the 13 PCGs began with typical ATN codons (five ATG, two ATA, and six ATT). Among them, 11 PCGs terminated with TAA, only *nad4* and *nad5* with incomplete stop codon TA and T, respectively. All the 22 tRNAs, ranging from 59 to 72 bp in length, had the typical cloverleaf structure, except for *trnS1*, whose dihydrouridine (DHU) arm formed a simple loop. The absence of the DHU arm in *trnS1* was found in the mitochondrial genomes existed in most insects (Wolstenholme 1992). The two rRNA genes, *rrnL* and *rrnS*, were 1310 bp and 754 bp in length, respectively. The control region was 3149 bp in length with 96.0% A + T content.

So far, there are 16 bumblebee species, whose mitochondrial genomes have been reported. These species including *B. filchnerae* in this study belong to nine subgenera: *Alpigenobombus*, *Bombus*, *Mendacibombus*, *Megabombus*, *Melanobombus*, *Psithyrus*, *Pyrobombus*, *Sibiricobombus*, and *Thoracobombus*. To confirm the phylogenetic relationships among nine subgenera of the genus *Bombus*, we performed a maximum-likelihood (ML) analysis based on the best-fitting substitution model of TIM+F+I+G4 with 1000 bootstrap replicates. The concatenated nucleotide sequences of 13 PCGs from 19 Apidae species including geographic populations of *Bombus terrestris* and *Bombus hypocrite* were used to construct the phylogenetic tree. Two honeybee species *Apis cerana* (accession number: GQ162109.1) and *Apis mellifera intermissa* (accession number: KM458618.1) were used as outgroups. The results indicated that *B. filchnerae* showed the closest relationship with *B. pascuorum*. Both of the species were clustered into a clade of subgenus *Thoracobombus* (Figure 1). The phylogeny tree also demonstrated clear relationships among the bumblebee subgenera, which were consistent with previous phylogenetic studies of bumblebees (Zhao et al. 2019; Wang et al. 2020). The mitochondrial genome of *B. filchnerae* reported in this study will provide more essential molecular data for further phylogenetic studies related to bumblebees and other pollinators.

**Figure 1.**
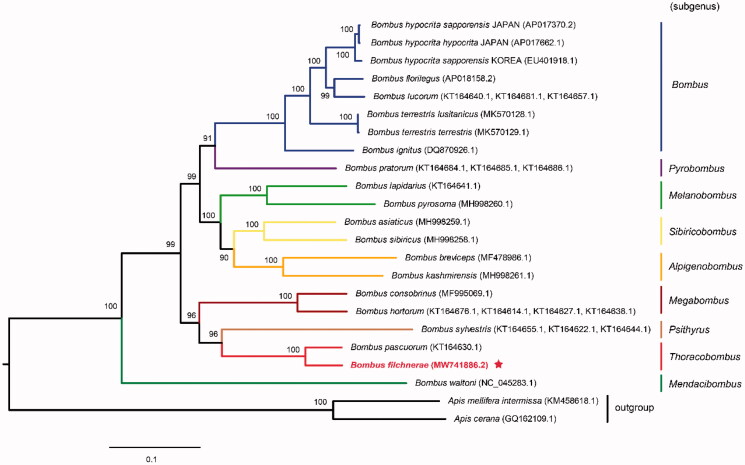
Maximum-likelihood tree showing phylogenetic relationships of *Bombus filchnerae* and other 19 Apidae species based on TIM+F+I + G4 model, using concatenated nucleotide sequences of 13 protein-coding genes. Numbers above or below nodes indicated the bootstrap support values estimated with 1000 replicates. The subgeneric names and outgroup related to phylogenetic analysis were depicted at right side. The nine subgenera included *Alpigenobombus*, *Bombus*, *Mendacibombus*, *Megabombus*, *Melanobombus*, *Psithyrus*, *Pyrobombus*, *Sibiricobombus*, and *Thoracobombus*. The newly sequenced mitogenome was highlighted by the star.

## Data Availability

The genome sequence data that support the findings of this study are openly available in GenBank at https://www.ncbi.nlm.nih.gov under the accession number MW741886.2. The associated BioProject, SRA, and Bio-Sample numbers are PRJNA714801, SRR13975265, and SAMN18318457, respectively.
